# The perceptions of midwives regarding audit and feedback on the use of the partogram at Vhembe District of Limpopo Province, South Africa

**DOI:** 10.4102/curationis.v40i1.1770

**Published:** 2017-08-31

**Authors:** Thanyani G. Lumadi

**Affiliations:** 1 Department of Health Studies, University of South Africa, South Africa

## Abstract

**Background:**

Audit and feedback is regarded as the cornerstone of clinical teaching to guarantee good practice and to correct poor performance. Feedback given to health professionals assists in narrowing the gap between the actual and the desired information. The findings of the research study on perceptions of midwives on audit and feedback highlighted aspects that needed improvement to address challenges on the use and documentation of the partogram.

**Objectives:**

The objectives of this article were to explore and describe the perceptions of midwives on auditing of the partogram by health professionals and to explore and describe the perceptions of midwives on the feedback that was given after audit was done.

**Method:**

A qualitative, exploratory and descriptive study was conducted to answer the two research objectives. Semi-structured face-to-face interviews were conducted with 17 midwives who were working in the labour wards of three hospitals. Eight steps of qualitative data analysis as indicated by Tesch were used to analyse the data.

**Results:**

The findings revealed that auditing and feedback is sometimes done by midwives themselves, midwives’ managers and district managers. Audit is done monthly or on a daily basis and sometimes inconsistently because of shortage of staff. Challenges indicated were lack of knowledge on the use of the partogram and lack of encouragement and praise when documentation was done correctly and that emphasis was mostly placed on negative aspects.

**Conclusion:**

The findings revealed that auditing and feedback and in-service education is done at the three hospitals, although challenges such as inconsistency in auditing because of shortage of staff, lack of knowledge on partogram use and on principles of giving feedback were highlighted.

## Introduction

Approximately 99% of 216 maternal deaths per 100 000 live births in developing countries were reported by World Health Organization (WHO), which were mostly because of obstructed and prolonged labour (WHO 2015). The increase of maternal and perinatal mortality rates led to the launch of the partogram by the Safe Motherhood Initiative in 1887 (Fraser, Cooper & Nolte 2006:953). A partogram is regarded as a valuable tool that can be used to monitor progress of labour and to identify when interventions are necessary in order to reduce complications (Lavender, Hart & Smyth 2013:2; Tayade & Jadhao 2012:256). Consequently, South Africa adopted the use of the WHO partogram in maternity labour wards in order to monitor foetal and maternal well-being (National Department of Health 2007:38, 39).

A standard for the use of the partogram is available in the Guidelines for Maternity Care in South Africa: a manual for clinics, community health centres and district hospitals (National Department of Health 2007:38, 39; 2015:44, 67). Hence the need to assess whether midwives are adhering to the guidelines as stipulated in the document. An audit can be described as a control measure to check whether midwives are documenting the findings on management of women in labour as prescribed on the guidelines. Midwives, ward managers and district managers audit the partogram and give feedback to midwives. Feedback is regarded as providing information on one’s performance and should be given to midwives either during or after auditing in order to improve midwifery practice. At Vhembe District, partogram is audited by ward managers, district managers and midwives using the evaluation tool that has been designed for the purpose.

### Problem statement

Challenges on the use of the partogram as a clinical practice guideline to improve intrapartum care in developing countries including South Africa were documented by many researchers (Markos & Bogale 2015:1; Ollerhead & Osrin 2014:1; Opoku & Nguah 2015:1). Gaps in recording and problems with the design of the tool were some of the challenges experienced by midwives. However, the use of audit and feedback in clinical practice can improve the use and documentation of the partogram (Pattison 2006).

In an effort to identify and address the challenges experienced by midwives in the use and documentation of the partogram, perceptions of midwives on auditing and feedback that was given by health professionals including ward managers and district managers were explored.

## Research aim

The aim of this study was to identify the challenges of auditing of the partogram and on feedback given to midwives by ward managers, district managers and midwives themselves.

## Research objectives

This study had the following research objectives:

- To explore and describe the perceptions of midwives on auditing of the partogram by health professionals.
- To explore and describe the perceptions of midwives on the feedback that was given after audit was done.

## Operational definition of concepts

- Audit: is defined as any summary of clinical performance of health care over a specified period of time (Flottorp et al. 2010:v). In this research study, audit would mean the health professionals including midwives themselves, ward managers and district managers evaluating the manner in which midwives have recorded the information they have obtained during monitoring of women who were in labour. Auditing can be done when the woman is still in the ward, and after the woman has been discharged.
- Feedback: is regarded as providing information in practical terms to health professionals who are either working as a team or as an individual, by reflecting on data that have been derived from their routine practice (Flottorp et al. 2010:v). In this research article, feedback would mean the information that is provided to midwives by their colleagues, by ward managers and by district managers during the auditing process or after auditing, which is a reflection on how they have recorded the data on the partogram.
- Health professionals: For the purpose of this research study, health professionals would mean midwives, ward managers and district managers.
- Midwife: is defined as a person who has successfully completed a midwifery education programme that is recognised in the country where it is located and who has acquired the requisite qualifications to be registered or legally licensed to practise midwifery (International Confederation of Midwives, ICM 2011). In this research article, a midwife would mean registered midwives working in the labour wards of the three hospitals selected for the study at Vhembe District in Limpopo Province.
- Partogram: is defined by English Oxford Living Dictionaries as a graphical representation of the progress of a confinement from the time when there is significant cervical dilatation, on which various physiological parameters of the mother and offspring are recorded periodically along with details of medical intervention. In this research article, partogram would mean the partogram approved by the South African National Department of Health as indicated on the Guidelines for Maternity Care in South Africa (National Department of Health 2007:38, 39).
- Perception: is defined by Macmillan English Dictionary online as a particular way of understanding or thinking about something.

## Significance of the study

The study provided valuable information that can be used to improve auditing and giving feedback to midwives who are using the partogram as a tool to monitor the progress of labour at the district and in other clinical settings. Correct use and documentation on the partogram may improve the quality of care rendered to women during labour, which may reduce maternal and foetal death.

## Literature review

Improving the use and documentation of the partogram is crucial in reducing complications during labour and births. Kidanto et al. (2012:1) found that audit in low-resource settings is applicable and can help to improve quality of care in obstetrics. In Uganda, Masika, Katongole and Govule (2015:37) found that improving partogram documentation and use requires constant monitoring and supervision and the review of practices of health workers involved in the use of the partogram.

Audit and feedback assist in prompting health care professionals to modify their practice when given performance feedback, which indicates that their performance is inconsistent with the desirable standard (Ivers et al. 2012:2). A key factor in achieving greater impact in giving feedback in clinical practice is the intensity of the feedback, the provision of the feedback by a senior person, face-to-face feedback to individuals and feedback that is given is combined with educational meetings (Pattison 2006).

According to Masika et al. (2015:37), regular feedback meetings on performance should be held and trends communicated to the staff so that there should be a sense of ownership. The other purpose of feedback is to encourage midwives to think about their performance and how they can improve it. Several barriers to giving feedback were highlighted by Cantillon and Sargeant (2008:1292) as a difficult task as those giving feedback may fear damaging their relationship with other staff members and may try to avoid undermining another person’s self-esteem. Corrective feedback may also be regarded as being critical especially in the presence of patients or other colleagues.

Cantillon and Sargeant (2008:1293) highlighted important aspects that need to be considered when giving feedback in clinical practice, which are clarity on the criteria that are being used to ensure understanding and cooperation, feedback given on specific behaviour rather than on general practice to avoid confusion and offering feedback at the time of an event or shortly afterwards when aspects are still fresh in an individual’s mind. Feedback should also lead to changes in the professionals’ thinking, behaviour and performance by encouraging individuals to verbalise perception of their own performance and to suggest ideas for improvement. According to Nicol (2007:2), feedback should help to clarify what good performance is, encourages self-learning, positive motivational beliefs and self-esteem, involves the candidate in decision making about audit process and policies and supports the development of learning by candidates and those assessing the practice.

## Research design and method

### Research design

A qualitative, exploratory and descriptive research design was used to explore and describe midwives’ perceptions on audit and feedback on the use of the partogram to monitor women during labour.

### Research method

#### Population and sampling

The population comprised 17 midwives working at three Vhembe District hospitals in Limpopo Province. Purposive sampling was used to select 17 midwives working at the three hospitals. Midwives were purposely selected based on the fact that they have been working in the maternity ward for at least six months and were willing to participate in the research study.

#### Data collection

The researcher conducted semi-structured, face-to-face interviews with midwives who agreed to be interviewed and who signed the consent form. Interviews were conducted between February and March 2012 in the private rooms of the selected hospitals. One of the questions asked to midwives was ‘How do you perceive the auditing and feedback on partogram use?’ Follow-up questions were asked in order to elaborate and clarify aspects. Data were tape recorded and then transcribed verbatim.

#### Data analysis

Tesch’s (1990) eight steps of qualitative data analysis as described in Creswell (1994:154–155) were followed. Co-coding was done and themes and sub-themes were developed.

#### Ethical considerations

Ethical approval was obtained from the Ethics Committee of the University of South Africa. The chief executive officers of the three hospitals and the Limpopo Provincial Government granted written permission to conduct the research. In order to ensure privacy, the interviews were conducted in private rooms. Witten consent was obtained from the midwives after thorough explanation of the nature and purpose of the research. Participants were informed that they can withdraw from the research study at any time if they wish to do so without any consequence. Codes instead of names were used on interview transcripts to protect the respondents’ identity.

#### Context of the study

The study was conducted in the maternity wards of the three hospitals found at Vhembe District in Limpopo Province, South Africa. The researcher selected the hospitals based on the fact that one was a regional (referral) hospital and two were district hospitals. Pregnant women visit the hospitals directly or as referrals from communities. Midwives and doctors are expected to use the partogram when attending to all women who are in labour.

## Trustworthiness

The researcher had good background knowledge in midwifery, nursing education and research methodology, which ensured that the research process was followed and relevant aspects in midwifery were considered (Speziale & Carpenter 2007:49). Co-coding was done electronically with an independent coder to ensure dependability. Credibility was established through prolonged engagement with midwives in the labour wards of the three hospitals. All data pertaining to the research study were kept safely by the researcher (Polit & Beck 2012:723). Triangulation of data collection methods was ensured by writing field notes and conducting literature review related to the partogram, audit and feedback in clinical settings.

## Findings and discussions

### Demographic information

All 17 participants were female and were working at the three selected hospitals. The hospitals were coded as A, B and C, whereas participants were coded as P1 or P2. In order to distinguish the hospital where the participant comes from, the participants were coded as PA-1 or PA-2.

The ages of participants ranges from 31 to 64 years. Seven out of 17 midwives completed a two-year diploma in midwifery training programme, 5 completed the four-year diploma in nursing and midwifery programme, 4 underwent one-year midwifery diploma course, 2 were advanced midwives and 1 had a degree in nursing. Fourteen of the 17 participants who were interviewed were not in managerial positions, whereas 3 were operational managers. Only one midwife had less than five years of experience, whereas the rest had experience of more than five years.

Years of experience, type of health facility and in-service training on partogram use were found by Wakgari, Tessema and Amano (2015:4) to be significantly associated with knowledge of partogram utilisation and that midwives at hospitals were more knowledgeable than those working at the clinics.

The findings of this research study are presented and discussed under two themes, namely perceptions of midwives on auditing of partograms by health professionals and perceptions of midwives on feedback given after auditing as displayed in Table 1.

**TABLE 1:** Themes and sub-themes on auditing and giving feedback on the use of the partogram.

Theme	Sub-theme
Perceptions of midwives on auditing of partograms by health professionals	Perception on auditing by ward managers Perceptions of midwives on auditing by midwives Perceptions of midwives on auditing by district managers Perceptions of midwives on auditors’ knowledge on the partogram
Perceptions of midwives on the manner in which feedback was given on the use of the partogram	Positive feedback on the use of the partogram Negative feedback on the use of the partogram

*Source:* Author’s own work

### Themes and sub-themes on auditing and giving feedback on the use of the partogram

#### Perceptions of midwives on auditing of partograms by health professionals

Midwives indicated that auditing is conducted by ward managers, midwives themselves and district managers. They further explained their views on auditors’ knowledge of the partogram and the audit and feedback process.

**Perceptions of midwives on auditing of partograms by ward managers:** Supervisors in the maternity wards are expected to audit patients’ records including the partogram. An operational manager in the labour ward indicated that she audits the partograms every morning:

‘Every morning when I come on duty, after taking report and there are women in labour, I [*manager*] check if the partograms are used and are being plotted correctly and give advice. It’s just that maybe during the day I don’t check it, but every morning for those who are in labour I quickly go through the partograms and see if they are plotted correctly.’ (PB-7, female, midwife)

One midwife indicated that the supervisor also checks the bed letters of the patient and how the partogram has been plotted.

‘The person who’s the overall supervisor also goes from patient to patient checking if the partogram is plotted incorrectly but even after the patient has delivered, our operational manager follows the bed letters and audits them and allocate the marks, so if somebody has not done it correctly, she will call you and discuss with you.’ (PA-1, female, midwife)

**Perceptions of midwives on auditing of partograms by midwives themselves:** Participants indicated that they used to audit the partograms themselves, but it is not possible to conduct such audits routinely because of shortage of staff. One of the participants said the following:

‘Previously it was an audit team from maternity ward that conduct the audit. We used to audit the partograms ourselves, three midwives come together and check the partograms, but sometimes we experience shortage of staff and we don’t do auditing once or even twice a month.’ (PA-3, female, midwife)

Health workers should be encouraged to routinely appraise and correct their own performance (Cantillon & Sargeant 2008:1293). Self-assessment is regarded as the first step in the feedback process and an important principle when the person is able to assess his or her personal performance and determine knowledge gaps (Algiraigri 2014:1; Anderson 2012:155). The study conducted by Fowler and Wilford (2016:e23) on formative feedback in clinical practice settings supports the findings of this study. Limited time and shortage of staff was indicated as a problem that leads to feedback that is not timely, not sufficient and specific. Another midwife indicated the following statements:

‘They [*managers*] encourage us to do auditing ourselves. I think this auditing is improving, because you know what, we have the strategy of evaluating the partogram, you check how it was plotted, may be two people, we write on our book, and we give ourselves the percentage according to what we have found, and then we show the person who is responsible that here you did wrong and here you did good, but this book started this month.’ (PA-1, female, midwife)

Experience in clinical practice is valuable for the process of providing feedback. Although self-assessment is important, Ibrahim et al. (2014:425) found out that interns may be unable to judge their own performance because of lack of knowledge and experience. Furthermore, Wakgari et al. (2015:4) found out that there is significant association between years of experience, on-job training and knowledge of partogram utilisation.

In this research study, it can be concluded that most of the midwives who participated in the study had sufficient years of experience of partogram utilisation including audit and feedback.

**Perceptions of midwives on auditing of partograms by district managers:** Midwives indicated that auditing is also done by district managers to ensure that recording of the partogram is done according to the stipulated standard. District managers were reportedly failing to visit the labour wards as midwives indicated that they have not seen them for two weeks. The following statements were indicative of the district managers’ visits to the hospital:

‘If the district managers come to assess us, they just give us feedback whether we are doing good or not. They used to come after two weeks, but now I just haven’t seen them, I don’t know what’s wrong.’ (PC-1, female, midwife)

**Perceptions of midwives on health professionals’ knowledge of the partogram and auditing and feedback:** Participants verbalised that some of the members of the auditing teams do not have thorough knowledge of the partogram and do not have experience in using it. Some of the statements verbalised were the following:

‘There are people who check the partograms but they are not used to the partogram.’ (PB-2, female, midwife)

‘They are not working with the partogram so they are not having experience.’ (PB-2, female, midwife)

‘Some of them (managers) don’t even enforce the use of the partogram because they have a problem, when they enforce its use, they will have to explain and they also do not understand.’ (PB-4, female, midwife)

Anderson (2012:154), conducted a study on giving feedback on clinical skills to students and concluded that despite faculty’s accumulating knowledge in the area for several decades, education on feedback is important. Clynes and Raftery (2008:405) identify inadequate supervisor training and education as barriers to effective feedback. Furthermore, Barendonk, Stalmeijer and Schuwirth (2013) indicate that expertise is important to performance assessment and that there is a great need for expertise development and practical experience of those who conduct auditing and feedback.

#### Perceptions of midwives on the manner in which feedback was given on the use of the partogram

Feedback is the report that is given to a midwife individually or in groups, which includes analysis of the data that are presented and providing suggestions for improvement in practice including the training needs of health workers.

**Positive feedback on the use of the partogram:** The feedback should take place in an atmosphere of support and challenge aiming at closing the gap between the current and the desired behaviour. Positive feedback increases motivation of performance. The importance of positive feedback and provision of education to those providing feedback was indicated by midwives:

‘Percentages are allocated when they audit so if you get lowest percentages, they show you where you went wrong.’ (PA-1, female, midwife)

‘Every Wednesday we have an in-service training on partogram, one can take maybe 10 bed letters of patients who are discharged and sit down and scrutinise jointly with the midwives from the clinic to assist each other, because at the end of each drill we give them the feedback and recommendations where we are lacking.’ (PC-6, female, midwife)

Feedback to health professionals is an essential part of improving a service. It is worth noting that midwives were awarded specific percentages for their performance. Wakgari et al. (2015:4) found in-service training to be mostly significant in improving partogram utilisation. Groves et al. (2015:3) recommend that all midwives and supervisors be given in-service training on partogram use and on auditing and giving feedback in a way that will be most useful to them. In addition, Yisma et al. (2013:1) recommends pre-service and on-job training on partogram utilisation to improve health professionals’ performance. Supervisors should pay attention and make time for providing frequent high-quality feedback (Plakht et al. 2013:20164).

**Negative feedback on the use of the partogram:** Some midwives were not satisfied with the feedback that was given after auditing by their managers.

‘We need positive feedback because is not only the mistakes that we do, if you can deliver ten woman and their babies are all alive, and you receive no pat on your back, but if one child dies or you have FSB [*Fresh Still Born*] it’s demoralising.’ (PA-3, female, midwife)

‘We don’t get positive feedback, all we get is those feedback of saying we got attitude.’ (PA-4, female, midwife)

In Kenyan hospitals, Nzinga et al. (2009:1) conducted a study on documenting the experiences of health workers expected to implement guidelines during an intervention study and found that one of the challenges among others was lack of systems to reinforce guidelines and to confront poor practice. Lack of recognition and appreciation was also indicated by midwives as some of the challenges that were experienced. Feedback should be given on what was directly observed and phrased in non-judgemental language to enhance understanding in communication (Cantillon & Sargeant 2008:1292). Ivers et al. (2012:2) indicate that effectiveness of audit and feedback depends on baseline performance and how the feedback has been provided. Negative feedback if not managed properly can demotivate individuals and cause deterioration of performance (Cantillon & Sargeant 2008:1293).

## Limitations of the study

The study was conducted at three selected hospitals in the district and the results cannot be generalised to other hospitals. Perceptions of other health professionals on using the partogram such as district managers, doctors and clinic midwives were not explored.

## Recommendations

Midwives indicated lack of consistency in auditing visits. It is recommended that planning on auditing and regular feedback be done and implemented to improve the use of the partogram.

Gaps in auditor’s knowledge on partogram and auditing and especially on giving feedback were highlighted. It is recommended that all supervisors and midwives be given in-service training on partogram use and on auditing and feedback in a way that will be most useful to the members.

The findings highlighted lack of time to conduct audit and feedback because of shortage of midwives. More staff in labour wards are needed in order to afford midwives ample time to perform self-assessment and provide feedback to each other. Adequate staffing would be helpful in ensuring that the partogram is used and plotted according to set standards.

Further research on auditing and comparing different ways of providing feedback in midwifery practice needs to be conducted.

## Conclusion

Auditing and feedback are viewed as important practices of improving the use of the partogram. It can be concluded that auditing of the partogram, giving feedback and in-service education is done at the selected hospitals. However, there is a need for consistency in auditing, planned periodic in-service education on the use of the partogram, training on strategies that are useful in giving feedback in clinical practice and provisioning more staff in the labour wards.

## References

[CIT0001] Algiraigri A.H, 2014, ‘Ten tips for receiving feedback effectively in clinical practice’, *Medical Education Online* 19(25141), 1–5. 10.3402/meo.v19.25141 PMC4116619 25079664

[CIT0002] Anderson P.A.M, 2012, ‘Giving feedback on clinical skills: Are we starving our young?’, *Journal of Graduate Medical Education (Reviews)* 154–158, viewed 21 November 2016, from https://www.ncbi.nlm.nih.gov/pmc/articles/PMC3399605/pdf/i1949-8357-4-2-154.pdf 10.4300/JGME-D-11-000295.1 PMC3399605 23730434

[CIT0003] Barendonk C., Stalmeijer R.E. & Schuwirth L.W.T, 2013, ‘Expertise in performance assessment: Assessors’ perspectives’, *Advances in Health Sciences Education* 18, 559–571, viewed 21 November 2013, from https://www.ncbi.nlm.nih.gov/pmc/articles/PMC3767885/pdf/10459_2012_Article_9392.pdf 22847173 10.1007/s10459-012-9392-x PMC3767885

[CIT0004] Cantillon P. & Sargeant J, 2008, ‘Teaching rounds: Giving feedback in clinical settings’, *British Medical Journal* 337(7681), 1292–1294. 10.1136/bmj.a1961 19001006

[CIT0005] Clynes M.P. & Raftery S.E.C, 2008, ‘Feedback: An essential element of student learning in clinical practice’, *Nurse Education in Practice* 8(1), 405–411. 10.1016/j.nepr.2008.02.003 18372216

[CIT0006] Creswell J.W, 1994, *Research design: Qualitative and quantitative approaches*, Sage, Thousand Oaks, CA.

[CIT0007] English Oxford Living Dictionaries, viewed 16 November 2016, from https://en.oxforddictionaries.com/definition/partogram

[CIT0008] Flottorp S.A., Jamtvedt G., Gibis B. & Mckee M, 2010, ‘Using audit and feedback to health professionals to improve the quality and safety of healthcare’, viewed 16 November 2016, from http://www.euro.who.int/__data/assets/pdf_file/0003/124419/e94296.pdf

[CIT0009] Fowler P. & Wilford B, 2016, ‘Formative feedback in the clinical practice setting: What are the perceptions of student radiographers?’, *Radiograph* 22(2016), e16–e24. 10.1016/j.radi.2015.03.005

[CIT0010] Fraser D.M., Cooper M.A. & Nolte A.G.W, 2006, *Myles textbook for midwives*, African edn, Elsevier, London.

[CIT0011] Groves M., Mitchell M., Henderson A., Jeffery C., Kelly M. & Nulty D, 2015, ‘Critical factors about feedback: “They told me what I did wrong; but didn’t give me any feedback”’, *Journal of Clinical Nursing* 24(11–12): 1737–1739. 10.111/jocn.12765 25694175

[CIT0012] Ibrahim J., McPhail M., Chadwick L. & Jeffcon S, 2014, ‘Interns’ perceptions of performance feedback’, *Medical Education* 48(1), 417–429. 10.1111/medu.12381 24606625

[CIT0013] International Confederation of Midwives (ICM), 2011, viewed 10 November 2016, from http://www.internationalmidwives.org/assets/uploads/documents/Definition%20of%20the%20Midwife%20-%202011.pdf

[CIT0014] Ivers N., Jamtverdt G., Flottorp S., Young J.M., Odgaard-Jensen J., French S.D. et al., 2012, ‘Audit and feedback: Effects on professional practice and health care outcomes’, *Cochrane Database of Systematic Review* 13(7), viewed 16 November 2016, from http://onlinelibrary.wiley.com/doi/10.1002/14651858.CD000259.pub3/epdf/standard 10.1002/14651858.CD000259.pub3 PMC11338587 22696318

[CIT0015] Kidanto H.L., Wangwe P., Kilewo C.D., Nystrom L. & Lindmark G, 2012, ‘Improved quality of management of eclampsia patients through criteria audit at Muhimbili National Hospital, Dar es Salaam, Tanzania. Bridging the quality gap’, *Pregnancy and Childbirth* 12(134), 1–7, viewed 18 May 2017, from https://bmcpregnancychildbirth.biomedcentral.com/articles/10.1186/1471-2393-12-134 23170817 10.1186/1471-2393-12-134 PMC3542082

[CIT0016] Lavender T., Hart A. & Smyth R.M.D, 2013, *Cochrane Database of Systematic Review* 7, viewed 16 November 2016, from http://onlinelibrary.wiley.com/doi/10.1002/14651858.CD005461.pub4/epd 10.1002/14651858.CD005461.pub4 23843091

[CIT0017] Macmillan English Dictionary Online, viewed 16 November 2016, from http://www.macmillandictionary.com/dictionary/british/perception

[CIT0018] Markos D. & Bogale D, 2015, ‘Documentation status of the modified World Health Organization partogram in the public Health Institutions of Bale zone, Ethiopia’, *Reproductive Health* 12(81), 1–6. 26336094 10.1186/s12978-015-0074-z PMC4558972

[CIT0019] Masika M.A., Katongole S.P. & Govule P, 2015, Improving Partogram documentation and use by health workers of Bwera Hospital: A process improvement research’, *International Journal of Nursing and Health Research* 2(4), 37–45.

[CIT0020] National Department of Health, 2015, *Guidelines for maternity care in South Africa: A manual for clinics, community centres and district hospitals*, 4th edn, Pretoria, National Department of Health, viewed 16 May 2016, from https://www.health-e.org.za/wp-content/uploads/2015/11/Maternal-Care-Guidelines-2015_FINAL-21.7.15.pdf

[CIT0021] National Department of Health, 2007, *Guidelines for maternity care in South Africa: A manual for clinics, community centres and district hospitals*, 3rd edn, Pretoria, National Department of Health, viewed 16 May 2016, from http://www.ais.up.ac.za/health/blocks/block9/Maternal%20Guidelines%202007.pdf

[CIT0022] Nicol D, 2007, ‘Principles of good assessment and feedback: Theory and practice’, from the REAP International Online Conference on Assessment Design for Learner Responsibility, 29th–31st May 2007, viewed 10 October 2016, from http://www.reap.ac.uk

[CIT0023] Nzinga J., Mbindyo P., Mbaabu L., Warira A. & English M, 2009, ‘Documenting the experiences of health workers expected to use guidelines on an intervention study in Kenyan hospitals’, *Implementation Science* 4(44), 1–9. 19627591 10.1186/1748-5908-4-44 PMC2726115

[CIT0024] Ollerhead E. & Osrin D, 2014, ‘Barriers to and incentives for achieving partogram use in obstetric practice in low and middle income countries: A systematic review’, *BMC Pregnancy and Childbirth* 14(281), 1–7. 25132124 10.1186/1471-2393-14-281 PMC4147181

[CIT0025] Opoku B.K. & Nguah S.B, 2015, ‘Utilisation of the modified WHO partograph in assessing the progress of labour in a metropolitan area in Ghana’, *Research Journal of Women’s Health* 2(2), viewed 16 November 2016, from http://www.hoajonline.com/journals/pdf/2054-9865-2-2.pdf

[CIT0026] Pattison R.C, 2006, *Audit and feedback effects on professional practice and health-care outcomes: RHL commentary* (last revised 15 December 2006), The WHO Reproductive Health Library, World Health Organization, Geneva, viewed 12 October 2016, from http://apps.who.int/rhl/effective_practice_and_organizing_care/rpcom2/en/

[CIT0027] Plakht Y., Shiyovich A., Nusbaum L. & Raizer H, 2013, ‘The association of positive and negative feedback with clinical performance, self-evaluation and practice contribution of nursing students’, *Nurse Education Today* 33(1), 1264–1268. 10.1016/j.nedt.2012.07.017 22889579

[CIT0028] Polit D.F. & Beck C.T, 2012, *Nursing research: Generating and assessing evidence for nursing practice*, 8th edn, Williams and Wilkins, Philadelphia, PA.

[CIT0029] Speziale H.J.S. & Carpenter D.R, 2007, *Qualitative research in nursing: Advancing the humanistic imperative*, 4th edn, William and Watkins, Crawfordsville, IN.

[CIT0030] Tayade S. & Jadhao P, 2012, ‘The impact of use of modified WHO partograph on maternal and perinatal outcome’, *International Journal of Biomedical and Advance Research* 03(4), 256–262. 10.7439/ijbar.v3i4.398

[CIT0031] Wakgar N., Tessema G.A. & Amano A, 2015, ‘Knowledge of partogram and its associated factors among obstetric care providers in North Shoa Zone, Central Ethiopia: A cross sectional study’, *Research Notes* 8, 407, viewed 21 November 2016, from https://www.ncbi.nlm.nih.gov/pmc/articles/PMC4558760/pdf/13104_2015_Article_1363.pdf 10.1186/s13104-015-1363-x PMC4558760 26337684

[CIT0032] World Health Organization (WHO), 2015, *Trends in maternal mortality: 1990 to 2015: Estimates by WHO, UNICEF, UNFPA, World Bank Group and the United Nations Population Division*, viewed 10 October 2016, from http://www.who.int/reproductivehealth/publications/monitoring/maternal-mortality-2015/en/

[CIT0033] Yisma F., Dessalegn B., Astatkie A. & Fesseha N, 2013, ‘Knowledge and utilisation of partograph among obstetric care givers in public health institutions of Addis Ababa, Ethiopia’, *Bio Med Central Pregnancy and Childbirth* 13(17), 1–9, viewed 21 November 2016, from http://www.biomedcentral.com/1471-2393/13/17 10.1186/1471-2393-13-17 PMC3552695 23331626

